# Applications and Challenges for Use of Cell-Penetrating Peptides as Delivery Vectors for Peptide and Protein Cargos

**DOI:** 10.3390/ijms17020185

**Published:** 2016-01-30

**Authors:** Mie Kristensen, Ditlev Birch, Hanne Mørck Nielsen

**Affiliations:** Section for Biologics, Department of Pharmacy, Faculty of Health and Medical Sciences, University of Copenhagen, Universitetsparken 2, DK-2100 Copenhagen, Denmark; mie.kristensen@sund.ku.dk (M.K.); ditlev.birch@sund.ku.dk (D.B.)

**Keywords:** cell-penetrating peptides, delivery vectors, peptide and protein drugs, drug delivery, biological barriers

## Abstract

The hydrophilic nature of peptides and proteins renders them impermeable to cell membranes. Thus, in order to successfully deliver peptide and protein-based therapeutics across the plasma membrane or epithelial and endothelial barriers, a permeation enhancing strategy must be employed. Cell-penetrating peptides (CPPs) constitute a promising tool and have shown applications for peptide and protein delivery into cells as well as across various epithelia and the blood-brain barrier (BBB). CPP-mediated delivery of peptides and proteins may be pursued via covalent conjugation of the CPP to the cargo peptide or protein or via physical complexation obtained by simple bulk-mixing of the CPP with its cargo. Both approaches have their pros and cons, and which is the better choice likely relates to the physicochemical properties of the CPP and its cargo as well as the route of administration, the specific barrier and the target cell. Besides the physical barrier, a metabolic barrier must be taken into consideration when applying peptide-based delivery vectors, such as the CPPs, and stability-enhancing strategies are commonly employed to prolong the CPP half-life. The mechanisms by which CPPs translocate cell membranes are believed to involve both endocytosis and direct translocation, but are still widely investigated and discussed. The fact that multiple factors influence the mechanisms responsible for cellular CPP internalization and the lack of sensitive methods for detection of the CPP, and in some cases the cargo, further complicates the design and conduction of conclusive mechanistic studies.

## 1. Introduction

The increasing occurrence of life-threatening and serious debilitating diseases demands effective treatment with highly potent and specifically acting molecules, such as therapeutic peptides and proteins, which comprise an increasing number of new drugs in the pipeline. However, the hydrophilic nature of peptides and proteins renders them impermeable to the lipidic membranes surrounding the individual cells and thus is crucial for the maintenance of homeostasis and as protection against invasion by harmful microorganisms and molecules. Thus, for therapeutic peptides and proteins having an intracellular target, a strategy that facilities permeation across the plasma membrane is essential for successful delivery to the target. Such a membrane permeation-enhancing strategy may also be applicable for drugs dosed via a non-injectable route of administration (e.g., oral, nasal, pulmonary), where cells organized in a tight epithelium must be traversed in order to reach the systemic circulation and subsequently a target receptor. Finally, peptide or protein-based therapeutics acting on the brain may likewise benefit from a formulation strategy enabling passage of the otherwise impermeable endothelial cells constituting an essential part of the blood-brain barrier (BBB). 

Cell-penetrating peptides (CPPs) comprise a family of promising delivery vectors, which traverse cellular membranes when present in non-toxic submicromolar concentrations and that in a concentration-dependent manner, thereby making them suitable as vectors for drug delivery. The CPPs are typically not exceeding 30 residues in length and often carry a positive charge, which facilitates electrostatic interactions with negatively charged cell-surface constituents, such as the glycosaminoglycans (GAGs) and sialic acids, as an initial step prior to membrane translocation. The history of CPPs goes back to 1988, where Frankel & Pabo discovered that the Human Immunodeficiency Virus (HIV) Trans-activator of transcription (Tat) protein possessed the ability to translocate across cellular membranes [1]. In 1991, Joliot *et al.* demonstrated effective cellular uptake of the *Drosophila* antennapedia Homeodomain protein, and the peptide sequence responsible for membrane permeation was a few years later narrowed down to the third helix of the full-length protein, which today is referred to as penetratin [2]. Since the discovery of Tat and penetratin, a number of peptides have been added to the still growing family of CPPs, which comprises classes of cationic, amphipathic, hydrophobic, and anionic CPPs, being either naturally derived, designed, or chimera sequences. In addition to the efforts spent on the discovery of new CPPs, various strategies have been exploited to improve the efficiency of already known CPPs, either via improving their resilience to enzymatic degradation or by enhancing their membrane-penetrating propensity. These strategies include changing amino acid stereochemistry from l to d and the inclusion of β or γ-amino acids [3] as well as non-primary amino acids [4]. In addition, cyclic [5] and branched CPPs [6] have been developed for these purposes. 

To date, CPPs have been successfully applied as delivery vectors for intracellular delivery of a variety of cargo molecules and delivery vehicles counting imaging agents [7], small-molecule drugs [8], liposomes [9], and biopharmaceuticals including oligonucleotides [10], peptides and proteins [11]. Moreover, the CPPs have shown to be applicable for transepithelial [12] and transendothelial [13] delivery of therapeutic peptides and proteins.

In addition to their application as inert vectors for delivery of cargo molecules, an emerging concept is the dual-acting CPPs, which are both membrane permeating and bioactive. Within this context, studies have demonstrated that, in addition to being cell-penetrating, selected CPPs are able to safely modulate the intestinal paracellular barrier [14,15], to act as neuroprotectants [16], to or induce apoptosis in cancer cells [17].

The scope of the present review will be applications of the CPPs as transport vectors for the delivery of peptides and proteins, and studies within the fields of CPP-mediated delivery across cellular membranes, including epithelia and the BBB, will be highlighted. In addition, the choice of formulation approach, mechanism of membrane permeation, and limitations in the use of CPPs as delivery vectors will be discussed.

## 2. Formulation Approach: Covalent Conjugation or Physical Complexation

Two approaches are generally applied when CPPs are employed as delivery vectors: covalent conjugation or physical complexation. Both approaches have their pros and cons, and which is the most appropriate choice for a specific CPP-cargo drug delivery system (DDS) relates to the physicochemical and the biochemical properties of both the CPP and its cargo.

Covalent conjugation of a CPP to a cargo peptide or protein ensures an inherent proximity of the CPP to its cargo and may be achieved chemically via e.g., disulfide bonds [18,19], amine bonds [20], or specific linkers [21] that facilitate release of the cargo when internalized into the cell. Alternative to chemical synthesis, an expression host, such as *E. coli* or *Saccharomyces*, may be employed to produce the CPP-cargo conjugate depending on the feasibility of production by chemical synthesis, which highly relates to the total number of amino acids in the CPP-fused drug as well as its folding complexity [22,23,24,25]. Using the latter methodology opens up for the production of larger and more complex proteins than is possible by chemical synthesis. However, conjugation of a CPP to a cargo may negatively affect the potency of the latter [23] as well as the cell-penetrating propensity of the former. If the potency is affected, use of a cleavable linker might constitute the basis of a prodrug strategy with the CPP constituting the pro-moiety. The covalent conjugation approach is typically laborious and likely involves optimization of protocols for every CPP-cargo conjugate.

On the other hand, physical complexation via electrostatic and/or hydrophobic interactions of a CPP with a cargo peptide or protein is easily obtained by simple bulk-mixing. This approach allows flexibility in the applied CPP-to-cargo molar mixing ratio, whereas, by using the covalent conjugation approach, access to terminal or intra-sequential binding sites is decisive for the possible carrier-to-cargo ratio. Whether non-covalent interactions will actually form between the CPP and its cargo is dependent on the physicochemical properties of both the CPP and the cargo molecules as well as the formulation principles applied [26,27]. In addition, a pool of poorly defined structures varying in size and composition typically appears after simple bulk-mixing. One may apply controlled mixing by the use of a microfluidic system in order to obtain low polydispersity, thereby enabling proper characterization of the complex. On the contrary, covalently conjugating of the CPP to its cargo results in a well-defined molecule, which constitutes a novel active pharmaceutical ingredient (API), thus demanding extensive efficacy and safety assessment studies.

## 3. Peptide and Protein Cargos Successfully Delivered by Cell-Penetrating Peptides

**Figure 1:**
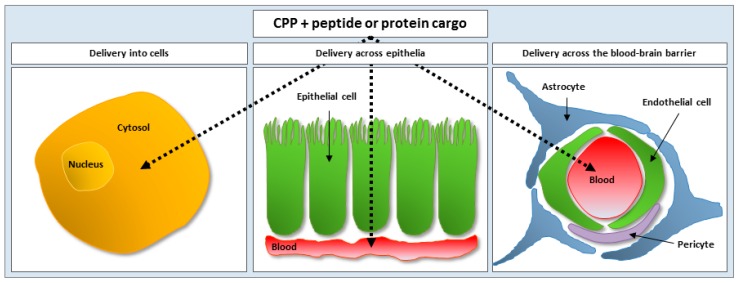
Applications for cell-penetrating peptides for the delivery of peptides and proteins into cells and across epithelia and the blood-brain barrier. Dashed arrows specifies target delivery site being in the cytosol or reached by systemic circulation.

Ten years after the discovery of the Tat peptide, its potential as a vector for the delivery of proteins into cells was demonstrated *in vitro* [28]. The following year, the effect was confirmed *in vivo*, as β-galactosidase was distributed to various tissues, including the brain, subsequent to intraperitoneal (IP) injection of a Tat-β-galactosidase fusion protein to mice [29]. Since then, CPP-mediated delivery has been applied to target numerous diseases (e.g., cancer, diabetes, and ischemia) (Table 1) by exploiting their ability to successfully act as vectors for delivery of therapeutic peptides and proteins into cells as well as across epithelia and the BBB (Figure 1).

### 3.1. Cell-Penetrating Peptides as Vectors for Intracellular Peptide and Protein Delivery

CPP-mediated delivery of peptides and proteins has been successfully applied to target cancer, where the tumor suppressor p53 is a prominent target, as mutations in the gene encoding for p53 is responsible for more than 50% of cancer incidents. In order to restore the p53 function, a number of p53-derived peptides have been tested and conjugated to CPPs in order to facilitate cellular uptake. This includes intra-peritoneal injection (IP) of a Tat-conjugated all-D retro-inverso (ri)-p53 [30] and an all-D undecaarginine (R11)-conjugated p53-ri-hemagglutinin-2 (HA-2) [31] to a peritoneal carcinomatosis mouse model, both of which positively affect apoptosis in the cancer cells, thereby prolonging survival of the treated mice. As is the case for p53, casein kinase 2 (CK2) is dysregulated in many human cancers, where it continuously supports proliferation and survival of the cancer cells. P15 is a peptide inhibiting the activity of CK2, and, when conjugated to Tat, P15 was internalized into cells from various tumor cell lines and apoptosis was then induced [32]. The anti-tumor effect of Tat-conjugated P15 was moreover demonstrated *in vivo*.

CPPs may also be employed to treat systemic and local inflammations resulting from invasion by pathogens or a chemical or physical injury. This was demonstrated in a study showing that the suppressor of cytokine signaling (SOC3) was able to internalize into immune cells and suppress inflammation in mice when conjugated to a CPP derived from the fibroblast growth factor 4 (FGF4). Moreover, this CPP-conjugated SOC3 was distributed to the liver where it protected the hepatocytes against liver apoptosis induced by virulence factors [33].

CPPs have also been applied to induce generation of pluripotent stem cells as a safer strategy than the classical methods involving introduction of viral genetic material, which are associated with great risk for mutagenesis and general genetic dysfunction; thus not being applicable to humans. Instead, a nonaarginine (R9) sequence was expressed conjugated to the C-terminal of proteins involved in cellular reprogramming (c-Myc, Sox2, Oct4, and Klf4), and the resulting fusion proteins successfully internalized into fibroblasts from human newborns, which subsequently transformed into pluripotent stem cells [34].

A large proportion of the reported CPP-mediated intracellular delivery of peptides and proteins is mediated via covalent conjugation of the CPP to its cargo. However, the far simpler physical complexation approach has also been successfully applied for the delivery of peptides and proteins into cells. As an example, a study demonstrated that the CPP Pep-1 was able to mediate intracellular delivery of co-administered peptide and protein cargos into cells from various cell lines, possibly due to the ability of Pep-1 to form hydrophobic interactions with the cargo peptide or protein [11].

### 3.2. Cell-Penetrating Peptides as Vectors for Delivery of Peptides and Proteins Across Epithelia and the Blood-Brain Barrier

A major obstacle to non-injectable administration of macromolecules or their delivery across the BBB relates to their inability to efficiently permeate epithelia or endothelia, respectively. However, prominent applications of the CPPs are their use as transport vectors for peptide and protein delivery across the intestinal [35], nasal [36], or pulmonary epithelia [37] as well as across the BBB [38].

Epithelia and endothelia constitute continuous tight barriers to uptake of macromolecules, as they are composed of individual polarized cells tightly interconnected by tight junctions (TJs), which only allow passage of water and small nutrients. Nevertheless, two overall routes may be exploited for delivery of peptides and proteins across epithelia and endothelia, namely, paracellular and transcellular permeation routes. Various classical chemical permeation enhancers, such as surfactants and bile salts, have been exploited in order to increase paracellular permeation of peptides and proteins. These, however, have often been found to act unspecifically, thus increasing the risk for adverse side-effects, and tend to negatively influence the cell membrane integrity and thus cellular morphology [39,40]. Therefore, novel, efficient and safe transport vectors to enhance the permeation of therapeutic peptides and proteins across epithelia and the BBB are highly welcome.

CPPs are promising candidates as safe and efficient vectors for transcellular transport of peptide and protein cargos. However, whether the CPPs are more specifically acting compared to the classical permeation enhancers may be discussed. In 2005, the first study reporting CPP-mediated transepithelial delivery of a cargo protein demonstrated that Tat, when covalently conjugated to insulin, significantly increased insulin permeation across Caco-2 cell monolayers [21]. Since then, additional CPPs have been employed as carriers for transepithelial delivery of therapeutic peptides and proteins, including polyarginines as carriers for insulin, glucagon-like peptide-1 (GLP-1), and gastrin [35,37,41,42], and penetratin as a carrier for insulin, interferon-β (INF-β), and GLP-1 [26,35,36,43]. However, not all CPPs, which have shown promising effects for intracellular delivery of a cargo are suitable for protein delivery across an epithelium or endothelium, as demonstrated in a study in which R8, penetratin, pVEC, and the RRL helix peptide were employed as carriers for systemic insulin delivery to rats following intestinal loop administration [35]. Only R8 and penetratin were able to significantly increase insulin permeation, whereas pVEC just had a minor positive influence on the plasma insulin concentration and the RRL helix peptide had no effect at all. Moreover, this study demonstrated that changing amino acid stereochemistry for all amino acids in the carrier peptide from the l-form to the d-form positively affected the R8-mediated insulin permeation. Further, a more recent study showed that an all-d-penetratin was superior to an all-l-penetratin as a carrier for transepithelial insulin delivery when administered to rats by oral gavage [43].

Penetratin is probably the most widely investigated CPP as a carrier for transepithelial delivery of peptides and proteins, and, in order to elucidate some sequence-specific properties responsible for its impressive potential, a range of penetratin analogues were synthesized and tested [44]. Among these was the Shuffle analogue with all residues except R and lysine (K) shuffled, and this peptide was able to significantly enhance insulin delivery in rats after nasal administration when compared to penetratin. A recent study moreover demonstrated that the effect of the Shuffle analogue could be improved further by interchanging the position of the N-terminal R residue with the C-terminal K residue resulting in the PenetraMax analogue [45]. Thus, the specific positioning of the individual amino acids in a CPPs sequence is highly important for its resulting carrier propensity.

Yet another application of the CPPs relates to their ability to act as vectors for delivery of therapeutic peptides and proteins to treat neurological disorders, as the delivery of macromolecules to targets in the brain is highly restricted by the BBB. In this context, CPPs have shown potential in overcoming this endothelial barrier, e.g., by conjugating Tat to Bcl-xl, which suppresses apoptosis in brain neurons. Subsequent to IP administration to mice, the Tat-Bcl-xl fusion protein was distributed into various parts of the brain and caused protection against ischemia [38]. Tat was moreover conjugated to the 9-amino acid C-terminal part of the *N*-methyl-d-aspartate receptor (NMDA) NR2B subunit (NR2B9c) [46] and the glial-derived neurotrophic factor (GDNF) for delivery into the brain to prevent ischemia [47].

**Table 1:** Selected peptide and protein cargos delivered by cell-penetrating peptides highlighting the formulation approach applied and the assays employed for studying tissue distribution or for verification of delivery.

Cell-Penetrating Peptide	Peptide or Protein Pargo	Formulation Approach	Assay	Ref.
*Delivery into cells*				
Tat	β-galactosidase	Covalent conjugation	Tissue distribution of β-galactosidase in mice following IP administration.	[29]
Tat	All-d-ri ^a^-p53	Covalent conjugation	Survival of peritoneal carcinomatosis mouse model following IP administration.	[30]
All-d-R11	p53~ri-HA-2 ^b^	Covalent conjugation	Survival of animals in a peritoneal carcinomatosis mouse model following IP administration.	[31]
Tat	P15	Covalent conjugation	Apoptosis in various tumor cell lines and regression of tumor size upon intratumoral injections to mice.	[32]
FGF4 ^c^-derived peptide [48]	SOCS3 ^d^	Covalent conjugation	Uptake into mouse macrophage cells and suppression of the production of inflammatory cytokines in mice following IP administration.	[33]
R9	c-Myc, Sox2, Oct4, Klf4	Covalent conjugation	Induction of fibroblasts from human newborn into pluripotent stem cells.	[34]
Pep-1	Various peptides and proteins	Physical complexation	Uptake of cargo peptide or protein in cells of various cell culture models.	[11]
*Delivery across epithelia and the blood-brain barrier*				
Tat	Insulin	Physical complexation	Insulin permeation across Caco-2 monolayers.	[21]
All-d-R8	Insulin, GLP-1 ^e^, gastrin	Physical complexation	Cargo plasma concentration following intestinal loop administration to rats.	[35]
Penetratin	Insulin, GLP-1, exendin-4	Physical complexation	Cargo plasma concentration following nasal or intestinal loop administration to rats.	[36]
All-d-penetratin	Insulin	Physical complexation	Blood glucose level following administration by oral gavage to rats.	[43]
Shuffle	Insulin	Physical complexation	Insulin plasma concentration following nasal administration to rats.	[44]
PenetraMax	Insulin	Physical complexation	Insulin plasma concentration following intestinal loop administration to rats.	[45]
Tat	Bcl-xl	Covalent conjugation	Brain distribution of Bcl-xl and reduction of cerebral infarction.	[38]
Tat	NR2B9c ^f^	Covalent conjugation	Brain concentration of NR2B9c in rats and reduction of cerebral infarction in mice following IP administration.	[46]
Tat	GDNF ^g^	Covalent conjugation	Brain concentration of GDNF and reduction of cerebral infarction following intravenous administration to mice.	[47]

^a^ Retro-inverso; ^b^ hemaglutinin-2; ^c^ fibroblast growth factor 4; ^d^ suppressor of cytokine signaling 3; ^e^ glucagon-like peptide-1; ^f^ 9-amino acid C-terminal part of the *N*-methyl-d-aspartate receptor (NMDA) NR2B subunit; ^g^ glial-derived neurotrophic factor.

## 4. Sequence-Specific Properties of Cell-Penetrating Peptides Influencing Their Membrane Permeation

The first step prior to cellular internalization of a CPP commonly involves interaction with plasma membrane constituents—usually through electrostatic interactions between positively charged R and K residues of the CPP and the negatively charged GAGs [49,50,51], sialic acids, or phospholipid headgroups [52] displayed on the cell surface. The affinity to such membrane constituents, however, differs according to the specific amino acid sequence in the CPP [53].

Much attention has been paid to the importance of R residue presence due to their guanidinium head group allowing for bidentate binding with negatively charged cell surface molecules, as opposed to the K residues, which donate only one positive charge via its ammonium group [54]. In fact, exchanging all R residues of the penetratin amino acid sequence with K residues (PenLys) completely abolished the ability of the molecule to act as a transport vector for co-administered insulin *in vitro* [26] and *in vivo* [44] when compared to the effect of the parent penetratin molecule. Thus, with respect to amino acid sequences of the CPPs, simply carrying a net positive charge due to the presence of K residues is not sufficient to act as a vector for the transepithelial delivery of co-administered insulin. However, high molecular weight polylysines (*M_W_* > 15 kDa) are able to enhance cellular uptake of protein cargo, as demonstrated as early as in 1965 by Ryser & Hancock, who reported increased albumin uptake when this molecule was applied together with polylysines [55]. On the other hand, when polyarginines are applied as CPPs, an optimal sequence length seems to be limited to 8–10 residues for mediating cellular uptake in a non-toxic manner [56,57]. Additionally, whether the polyarginine sequence is covalently conjugated to, or administered as a physical complex with its cargo, may influence the resulting toxicity of the CPP-cargo system. This was demonstrated in a recent study, showing that the covalent conjugation of an R9 sequence to the biologically active part of the parathyroid hormone which constitutes the N-terminal 34 amino acid residues (PTH(1-34)) negatively affected the cellular viability when applied to Caco-2 cell monolayers, whereas applying similar concentrations of PTH(1-34) physically complexed with equimolar amounts of R9 was not associated with any adverse effects [23].

In addition to the importance of cationic R residues in the CPP, multiple studies have demonstrated the importance of hydrophobic residues for the ability of CPPs to interact with and insert into the plasma membrane. Tryptophan (W) residues have been shown to improve the interaction with cell-surface-exposed GAGs, which are believed to be involved in the process of endocytic CPP uptake [58,59]. Furthermore, a direct positive correlation between the number of W residues in a basic CPP sequence and the binding affinity to GAGs in solution, with which they form stable aggregates, has been reported [53]. Further, not only the presence, but indeed also the specific positioning of W residues in a CPP sequence, influences the resulting efficiency in membrane permeation [60].

Thus, the primary amino acid sequence of CPPs, both in terms of individual residual properties as well as their specific positioning within the sequence, is of great importance for the resulting cell-penetrating propensity of the CPP. Moreover, the ability of some CPPs to adopt a well-defined α-helical or β-sheet secondary structure when encountering the cellular plasma membrane appears to positively correlate with the propensity to translocate across the membrane [61,62,63].

## 5. Mechanisms of Membrane Permeation of Cell-Penetrating Peptides

Much attention has been put into elucidating the mechanisms facilitating membrane permeation of the CPPs, but the puzzle is still not fully understood. Nevertheless, it is today generally accepted that both endocytosis and direct translocation contribute to membrane translocation of CPPs, though it was previously believed that only direct translocation was involved. However, since protocols involving the use of fixatives were shown to cause artefacts in membrane permeability [64], several studies have demonstrated endocytosis to be a mechanism of cellular uptake of CPPs [53,65,66,67].

It is important to note that whether the CPP exploits endocytosis or direct translocation across a membrane is dependent on multiple factors, such as the specific CPP sequence [68], the CPP concentration [69], the specific cell-type [70], and the state of cellular differentiation [71]. Moreover, the cargo [72,73,74], as well as the CPP-cargo formulation design being either covalent conjugation or physical complexation [23], and the conjugation of a label for detection [74,75], may be decisive for the mode of membrane permeation. Additionally, it is likely that multiple mechanisms are active simultaneously, again depending on the above mentioned factors, thereby further complicating the search for common conclusions on the mechanism responsible for cellular internalization of the CPPs and their cargos. To date, most mechanistic studies relate to the mechanisms by which the CPPs and their cargos are internalized into cells, thus not explaining how the CPPs (if at all) and their cargos are translocated across the epithelial and endothelial barriers. However, the mechanisms responsible for the initial membrane interaction and subsequent translocation across the apical plasma membrane likely share similarities with the mechanisms exploited for the CPP-mediated intracellular delivery of cargos.

**Figure 2:**
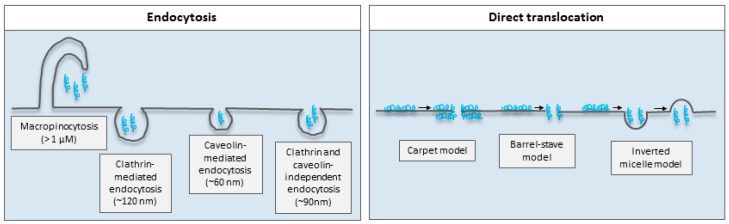
Simple illustration of mechanisms by which cell-penetrating peptides have been suggested to traverse the plasma membrane by endocytosis or direct translocation. Dimensions are obtained from [77]. Modified from [96] with permission from Elsevier. Blue helixes represent the CPPs, the arrow the uptake process subsequent to membrane interaction.

Phagocytosis and pinocytosis are two overall endocytic mechanisms, which involve energy-dependent formation of vesicles of various sizes. Phagocytosis, also referred to as “cell eating”, takes place in specialized scavenger cells, such as the macrophages, and is responsible for the engulfment of cell debris, bacteria, and other large particulate matter [76]. On the other hand, pinocytosis, or “cell drinking”, contribute to the control of cellular homeostasis and takes place in all cell types [77]. Pinocytosis is further segregated into the pathways macropinocytosis, clathrin-mediated endocytosis, caveolin-mediated endocytosis, or clathrin- and caveolin-independent endocytosis, all of which differ by the mechanism of vesicle formation as well as the resulting size of these [77,78] (Figure 2). Various studies have demonstrated cellular uptake of arginine-rich CPPs, such as Tat and polyarginines, by macropinocytosis for which membrane ruffling plays an important role [79,80]. In addition, uptake of a Tat-conjugated protein was shown to exploit caveolae-mediated endocytosis [65], whereas a Tat-conjugated fluorophore was demonstrated to be taken up via clathrin-mediated endocytosis [81], thereby underlining that the physicochemical properties of the cargo is highly relevant for the main mechanism exploited for the CPP-mediated cellular uptake.

Suitable methodologies to demonstrate endocytic uptake includes the use of microscopy to localize fluorescently tagged CPPs in the cytosol or in vesicular structures [82,83], and the specific active endocytic mechanism may be identified by the use of chemical inhibitors [84] or by the more recently developed methodology employing siRNA inhibition of individual endocytic mechanisms [85]. If an endocytic mechanism is highjacked by a CPP for intracellular delivery of its cargo, a successful outcome relates to the potential for endosomal escape before being directed to lysosomes for degradation or recycling of endosomes for transport back to the plasma membrane and subsequent extracellular release. A study demonstrated that cyclic CPPs facilitate membrane destabilization at the low pH (approximately 5.5) in endosomes, and are thus a strategy for enhancing endosomal escape [86]. On the other hand, if transepithelial or transendothelial CPP-mediated transport of a cargo drug is intended, the prominent criterion for efficient drug delivery relates to whether transcytosis follows the endocytic uptake. Transcytosis is exploited by various toxins subsequent to oral ingestion in order to transverse the epithelium for gaining access to the systemic circulation [87,88], but may also be the mechanism by which peptide and protein drugs are translocated across epithelia and endothelia when CPPs are employed as delivery vectors. 

As opposed to mediating endocytic cellular uptake, some CPPs are able to directly translocate across the plasma membrane in an energy-independent manner as demonstrated by the fact that CPP-uptake takes place when the temperature is lowered to 4 °C [89]. Different modes by which CPPs directly translocate across the plasma membrane have been proposed: the carpet and the barrel-stave models [90], the inverted micelle model [91], the electroporation-like model [92], and the counterion model [93] (Figure 2). Penetratin has in some studies been demonstrated to translocate across membranes via mechanisms explained by the carpet and the barrel-stave models, which both involve pore formation, but differ according to the orientation of the CPP relative to the membrane lipids [90]. With the inverted micelle model, electrostatic interactions between the CPP and negatively charged membrane phospholipids facilitates insertion into the membrane, after which interaction between hydrophobic CPP residues and the membrane core leads to changes in membrane curvature, resulting in the formation of vesicles in which the CPP is taken up. This mechanism was first demonstrated for penetratin in 1996 [91], *i.e.*, before it was well-known that fixation causes artefacts in membrane integrity. However, later studies have demonstrated the involvement of energy-independent vesicular CPP uptake [94,95] differing from endocytosis by the smaller size of the formed vesicles, as well as by the fact that this mechanism is active at 4 °C. The electroporation-like model [92] and the counterion model [93] involve either a local charge neutralization or the formation of a transient electrical field, respectively. The former mode of translocation has been used to explain uptake of penetratin into cells [92], whereas the latter is found relevant for the guanidinium-rich polyarginines [93].

If the CPP is intended to be applied as a vector for delivery of a cargo drug with a target site that must be reached via the systemic circulation upon non-injectable drug administration or for at drug having a target site in the brain, both the apical and basolateral membranes of the epithelial or endothelial cells, respectively, must be overcome. In this case, the steps of initial membrane interaction and subsequent translocation for cellular internalization are most likely similar to those involved for CPP-mediated intracellular delivery of a cargo drug. However, the importance of the presence of the CPP for exocytosis or direct translocation across the basolateral membrane subsequent to cellular internalization via the apical side of the plasma membrane is not known. In addition, paracellular delivery across the epithelium or endothelium may contribute to the net delivery of the cargo by directly affecting the opening and closure of the TJs via local high CPP concentrations [89] or indirectly through the binding to intracellular targets regulating the TJ dynamics [14].

## 6. Challenges for the Use of Cell-Penetrating Peptides as Delivery Vectors

For CPPs to be useful as vectors for the delivery of any therapeutic entity, several considerations have to be taken into account, e.g., that CPPs due to their peptidic nature are labile molecules prone to degradation following exposure to biological fluids such as blood and intestinal juice [97]. Additionally, available assays for sensitive detection and thereby tracking of CPPs in the biological matrices is limited mainly to fluorescence detection upon the use of fluorophore-conjugated CPPs. Fluorophore conjugation to short peptides inevitably will alter the physicochemical characteristics of the molecules and affect the membrane permeating propensity of the CPP [75]; thus detection of the non-labeled CPP would be favored. Unfortunately, the detection of non-labeled CPPs in general suffer from the lack of antibodies directed against such short peptides, which would allow for the development of e.g., enzyme-linked immunosorbent assays (ELISAs) and radioimmuno assays (RIAs). 

### 6.1. Stability of Cell-Penetrating Peptides

Despite great potential for CPPs as vectors for drug delivery, their use is limited by an inherent chemical instability making them prone to degradation by extracellular and intracellular enzymes. This will consequently lower the concentration of the CPP after exposure to biological matrices at the action site, and stabilized CPPs are thus commonly employed to slow down the degradation kinetics of the CPP. However, some degree of controlled biodegradability is a desirable feature of CPPs when exploited as delivery vectors, since biodegradability to some extent relates to biocompatibility and thus patient safety.

Several strategies have been employed for improving the CPP stability. One is to alter the stereochemistry by changing the amino acid stereochemistry by use of the unnatural d-form of one or more amino acids in the CPP sequence. This will prolong the CPP half-life in biological fluids, as observed in a recent study demonstrating that the half-life of an all-d-penetratin was markedly prolonged when compared to the half-life of l-penetratin subsequent to incubation with rat intestinal fluid [43]. Alteration of the amino acid stereochemistry may, however, negatively affect the resulting cell-penetrating propensity of the CPP and/or its ability to act as a delivery vector. A previous study reported the ability of l- and d-penetratin to mediate delivery of exendin-4, GLP-1, and INF-β across nasal and intestinal epithelia [36]. It was observed that d-penetratin increased the plasma concentration of INF-β to a greater extent than L-penetratin; however, the opposite was true for exendin-4 and GLP-1. Thus, changing CPP amino acid stereochemistry from l to d may improve the stability and membrane permeation of the CPP, but does not necessarily increase delivery of all cargo drugs.

Another strategy to enhance the chemical stability of CPPs is to modify the amino acid residues at specific sequential locations in order to reduce the access of proteases to cleavable sites. This approach has been applied for the generation of human calcitonin (hCT)-derived CPPs in which amino acid substitutions to d-phenylalanine or *N*-methyl-phenylalanine at specific locations significantly improved the resilience of the CPP to proteolytic degradation when incubated with HEK 293T cells as well as in human blood plasma, without compromising the cell-penetrating propensity of the CPP [98]. This strategy seems promising, but requires extensive knowledge of the degradation pattern of the CPP with identification of major cleavage sites, as previously investigated for the anionic sweet arrow peptide and the cationic penetratin, Tat, MPG family members [Pβ] and [Pα], as well as hCT-derived CPPs subsequent to incubation with epithelial cell culture models [99,100]. It should also be noted that the substitution of single non-natural residues in the amino acid sequence not only requires changes in the process of synthesis, but may also add significantly to the costs of the CPP.

Finally, one may prolong the CPP half-life via backbone stabilization by the inclusion of β- and γ-peptoid residues in the CPP sequence [101,102,103]. Backbone stabilization may, in addition to enhancing the chemical stability, also improve the physical stability, e.g., by the stabilization of the CPP structure though electrostatic interactions or by increasing the distance between residual side-groups with similar charges [103,104].

Regardless of which strategy is exploited to improve the stability of the CPP, the resulting molecule must be viewed as a novel excipient and therefore needs proper characterization and preclinical testing. Parameters, such as cellular toxicity and cell-penetrating propensity, may be altered, and a decrease in clearance rate can lead to side-effects and unwanted immune responses. Thus, extended safety and efficacy studies must be conducted before the implementation of the CPP as a delivery vector.

### 6.2. Detection of Cell-Penetrating Peptides and Their Cargo Peptide or Protein Drugs

Experimental work with peptides and proteins often implies the need for detection of these in submicromolar concentrations and in complex biological matrices, e.g., when CPPs are employed as delivery vectors for peptide and protein cargo into cells as well as across epithelia or endothelia. Due to the low permeation of peptides and proteins across biological barriers, methods for the detection of submicromolar concentrations are necessary. For the detection of classical therapeutic cargos, such as the PTH(1-34) and insulin, reliable and highly sensitive ELISA assays are commercially available [23,26]. However, this is not the case for all relevant peptide and protein cargos, and a general lack of specific antibodies hinders the development of such assays for detection. Additionally, the generation of specific antibodies directed against CPPs is highly challenging due to their short sequence and are to date not commercially available. Therefore, researchers rely on indirect strategies for detection and tracking of the CPP, such as via conjugation of the CPP to a fluorophore [7,74], metal ions [105], the incorporation of radioactive isotopes [106], or even a combination of the latter two [107].

Fluorophore conjugation is by far the most commonly used strategy for the detection of CPPs, which makes it possible to study the cellular uptake and distribution by, e.g., confocal laser scanning microscopy, or for the quantification of cellular uptake or transepithelial translocation using flow cytometry or fluorescence-based liquid chromatography, respectively. However, fluorophore conjugation changes the physicochemical properties of the CPP in terms of, e.g., size and hydrophilicity [75,105], thereby possibly affecting the resulting cell-penetrating propensity, cellular toxicity and, if covalently conjugated to a cargo drug, the potency of this. Utilizing metal ions or radioactive isotopes for labeling may likewise affect the physicochemical properties of the CPP [108]; however, given their small size, the effect is likely to be minor as demonstrated in a study comparing fluorophore-labeling and selenium-labeling of penetratin with non-labeled penetratin in terms of the resulting hydrodynamic radius, secondary structure, cellular uptake, and general effect on membrane leakage [105]. Importantly, it must be realized that the detection of a label rather than the CPP may lead to false positive results since the label may be cleaved off the CPP during the uptake and processing of the CPP. In addition, the inherent desirable degradation of the CPPs will generate peptide fragments, some of which are labeled, with different physicochemical properties than the full-length CPP. However, once a suitable method for labeling is specified, quantification of the labeled fragments may be achieved by flow cytometry, inductive coupled plasma mass spectrometry or scintillation counting for fluorophores, metal ions, and radioactive labels, respectively. Of these, in the field of CPP research, only limited work has been published on the use of metal ions and radiolabeling. Radiolabeling is an expensive procedure, for which approved facilities must be accessible for preparation, storage, use, and analysis of the labeled peptide or protein. In addition, neither detection via the label, nor detection by the use of, e.g., ELISA, reveals whether the delivered CPP is intact and, importantly, biologically active, similarly to what must be acknowledged for the detection of the cargo. To achieve that kind of information for the cargo drugs, the availability of robust and reliable *in vivo* models is essential, either via a measurable physiological response, such as an increase in blood Ca^2+^ or a lowering of blood glucose subsequent to administration of PTH(1-34) or insulin, respectively, or by a therapeutic effect in a disease-specific model.

## 7. Future Perspectives

Since the discovery of the Tat peptide almost 30 years ago, the field of CPP research has significantly progressed. Novel and optimized sequences are continuously added to the growing family of CPPs, which have demonstrated potential for the delivery of various types of cargos—not only into cells, but also across epithelial and endothelial barriers. Multiple research groups invest significant efforts into understanding the mechanisms by which the CPP are able to translocate cell membranes. However, the fact that multiple factors influence the mechanism(s) exploited by the individual CPPs further complicate the picture, and each cargo and/or target cell or barrier likely to some extent need a tailor-made CPP.

Especially the cationic CPPs have shown great potential in the challenging field of peptide and protein delivery into cells as well as across epithelial barriers and the BBB both *in vitro* and *in vivo*. However, in order to significantly propel the field, a thorough understanding of the basic mechanisms leading to translocation of the CPP and its cargo is essential for further development of these as safe and functional delivery vectors for peptide and protein cargos. A proper mechanistic understanding must be obtained at several levels. Both with respect to the importance of intermolecular CPP-cargo interactions and parameters controlling these, and, if applied for transepithelial drug delivery, interaction with the mucus as well as how the cargo—with or without the CPP—is translocated across the basolateral membrane. The latter aspect also relates to the use of CPPs for the delivery of cargo across the BBB. Crucial to success in applying CPPs as carriers for peptide and protein drug delivery is, however, the development of sensitive and reliable analytical methods, which allow detection of minute amounts of unmodified CPP in complex biological media and robust *in vitro* models representing *in vivo* conditions as well as reliable *in vivo* models. To date, most *in vivo* studies exploiting the CPPs as carriers for peptide and protein delivery are performed with simple CPP-cargo formulations. However, the safe introduction of the CPPs as vectors *in vivo* requires the development of more advanced target-specific DDSs in order not merely to ensure efficient delivery and release of the CPP and its cargo at the target tissue, but also to avoid adverse side-effects due to off-target distribution of the CPP and its cargo.
